# Higher levels of plasma Adrenocorticotropic hormone (ACTH) are associated with lower suicidal ideation in depressed patients compared to controls and suicide attempters, independently from depression severity

**DOI:** 10.1016/j.cpnec.2024.100235

**Published:** 2024-04-26

**Authors:** Robertas Strumila, Aiste Lengvenyte, Linas Zdanavicius, Robertas Badaras, Edgaras Dlugauskas, Sigita Lesinskiene, Eimantas Matiekus, Martynas Marcinkevicius, Lina Venceviciene, Algirdas Utkus, Andrius Kaminskas, Tomas Petrenas, Jurgita Songailiene, Dalius Vitkus, Laima Ambrozaityte

**Affiliations:** a Department of Urgent and Post Urgent Psychiatry, CHU Montpellier, Montpellier, France; b Institute of Functional Genomics, CNRS, INSERM, University of Montpellier, Montpellier, France; c Clinic of Psychiatry, Institute of Clinical Medicine, Faculty of Medicine, Vilnius University, Vilnius, Lithuania; d Centre for Toxicology, Clinic of Anaesthesiology, Reanimatology and Critical Care Medicine, Institute of Clinical Medicine, Faculty of Medicine, Vilnius University, Vilnius, Lithuania; e Vilnius City Mental Health Center, Vilnius, Lithuania; f Centre for Family Medicine, Vilnius University Hospital Santaros Klinikos, Faculty of Medicine, Vilnius University, Vilnius, Lithuania; g Department of Human and Medical Genetics, Institute of Biomedical Sciences, Faculty of Medicine, Vilnius, Lithuania; h Centre of Laboratory Medicine, Vilnius University Hospital Santaros Klinikos, Vilnius, Lithuania; i Department of Physiology, Biochemistry, Microbiology and Laboratory Medicine, Institute of Biomedical Sciences, Faculty of Medicine, Vilnius University, Vilnius, Lithuania

**Keywords:** Suicidal ideation, Adrenocorticotropic hormone (ACTH), Hypothalamic-pituitary-adrenal (HPA) axis, Cortisol, Antidepressants, Major depressive disorder (MDD), Biomarkers, Inflammation (IL6 and TNF alpha), Stress response, Beck scale for suicide ideation/suicide severity index (BSS/SSI)

## Abstract

**Introduction:**

Suicidal ideation, an important risk factor for suicide attempts, has an unclear neurobiological basis and is potentially linked to the dysregulation of the hypothalamic-pituitary-adrenal (HPA) axis and immune-inflammatory systems. While inflammatory markers have been associated with suicide attempts and, to a lower extent suicidal ideation, the data on the role of a stress-response system is less robust, with most studies carried out with cortisol showing inconsistent results. The present study extends on the previous studies implicating stress-response and immune-inflammatory systems in suicidal thoughts and behaviours, focusing on the associations of several stress-response (adrenocorticotropic hormone (ACTH), cortisol, and dehydroepiandrosterone (DHEA)) and immune-inflammatory (C-reactive protein (CRP),interle ukin-6 (IL-6), and tumour necrosis factor-alpha (TNF-alpha)) with suicidal ideation severity in recent suicide attempters, patients with major depressive disorder, and non-psychiatric controls.

**Methods:**

This observational study included 156 adults from three Vilnius hospitals, recruited into one of the three groups in equal parts: recent suicide attempters, patients with major depressive disorder in current depressive episode, and non-psychiatric controls. Measures included the Hamilton Depression Rating Scale (HDRS-17) and the Beck Scale for Suicide Ideation/Suicide Severity Index (BSS/SSI), alongside sociodemographic data, alcohol, tobacco use, and morning blood samples, measuring plasma ACTH, cortisol, DHEA, CRP, and IL-6. Data were analysed with non-parametric tests, Kendall's tau correlation, and multivariate linear regression adjusted for confounders.

**Results:**

We found a negative correlation between the plasma ACTH levels and suicidal ideation severity (tau = −0.130, p = 0.033), which was driven by the patients with major depressive disorder (tau = −0.237, p = 0.031). Suicidal ideation severity was also negatively correlated with TNF-alpha (tau = −0.231; p < 0.001), positively correlated with IL-6 (tau = 0.154, p = 0.015), and CRP levels (tau = 0.153, p = 0.015), but no differences were observed in group-stratified analyses. The association between plasma ACTH levels and suicidal ideation severity in patients with major depressive disorder remained robust to adjustment for major confounders (adjusted for age, sex, education years, body mass index, smoking status, plasma CRP and PEth concentration (measuring chronic alcohol exposure), and antidepressant use) in the linear regression model (t = −2.71, p = 0.011), as well as additionally adjusting for depression severity (t = −2.99, p = 0.006).

**Discussion:**

The present study shows an association between plasma ACTH levels and suicidal ideation severity in patients with major depressive disorder, robust to adjustment for antidepressant use and depression severity. This finding highlights the potential role of ACTH, in elucidating the effects of stress and mental health disorders. Our findings underscore the importance of the HPA axis in the diagnosis and treatment of suicidal ideation in major depressive disorder and invite further research on interventions targeting this pathway.

## Highlights

• The study explored the link between ACTH and suicidal ideation, focusing on a lesser-known aspect of the HPA axis.
• ACTH levels negatively predicted with suicidal ideation, especially in depression group, but it was not related to depression severity.
• ACTH levels significantly predicted with suicidal ideation severity in psychiatric patients, even after accounting for confounders
• Only suicide attempters showed a positive correlation between cortisol and suicidal ideation, pointing to known discrepancies.
• The results emphasize the HPA axis's and ACTH role in understanding suicidal ideation's neurobiology

## 1 Introduction

Suicidal ideation is a significant risk factor for suicide and suicide attempts, and its early identification and management can prevent further progression towards completed suicide [1,2]. It is a common phenomenon in suicide attempters, and people with psychiatric illnesses, of which major depressive disorder (MDD) is the most common one, and less frequent, but still observed in the general population [3]

Although the neurobiological basis of suicidal ideation remains unclear, dysregulation of the hypothalamic-pituitary-adrenal (HPA) axis and immune-inflammatory systems have been implicated in the pathophysiology of suicidal thoughts and behaviours (STB) [[4], [5], [6]]. It has been proposed that the bidirectional association between cortisol cytokine regulation is involved in STB [7]. Furthermore, some studies have reported differences in some inflammatory, such as the acute phase reactant protein C-reactive protein (CRP) and interleukin (IL)-6, as well as some other less-studied cytokines, and HPA activity markers, such as blunted HPA axis activity between suicide attempters and depressed individuals or those considered at risk of suicidal behaviours without suicide attempt history [5,[8], [9], [10], [11], [12], [13]]. Meanwhile, increased immune-inflammation markers and HPA axis dysregulation have also been consistently associated with depression [14]. While studies show that the association between immune-inflammatory dysregulation and STB is at least partly independent of depressive symptoms, associations with SI seem to be less robust than with SA [6,10] and studies on HPA axis markers are even more inconsistent.

Cortisol, the primary glucocorticoid hormone released by the adrenal gland in response to stress, is one of the most widely studied biomarkers of the HPA axis dysregulation in patients with depression and STB [14,15]. However, research on the relationship between cortisol and STB was complicated by several factors. First, there have been inconsistent findings regarding cortisol levels in suicide attempters [16]. This inconsistency may be due to various factors such as small sample sizes, differences in diagnostic criteria, and variations in methods for measuring cortisol levels, including taking into account the marked variation of plasma cortisol levels throughout the day [16]. To make things more complex, in depressed patients, plasma cortisol levels can stay elevated throughout the day, further complicating the comparisons [17,18]. Such Inconsistencies have led to the stalling of HPA system-related marker studies in STB, possibly stalling the progress towards clinically useful biomarkers.

Besides immune markers and cortisol, another interesting option is to study the hormone that is tightly linked to cortisol and is involved in inflammation regulation, the adrenocorticotropic hormone (ACTH) [19,20]. Prolonged exposure to ACTH is sometimes used to produce a depressive phenotype in experimental animal models [21]. However, depression, and suicidal ideation, although tightly linked, are not the same thing [22]. To date, research on ACTH in patients with STB is scant and inconsistent [[23], [24], [25]]. One potential explanation for the inconsistent findings is the heterogeneity of suicidal ideation, which can range from passive thoughts about death to active planning and preparation for suicide [26]. Different levels of suicidal ideation may be associated with different patterns of HPA axis dysregulation [27]. Therefore, examining the relationship between ACTH and suicidal ideation across different levels of severity may provide a better understanding of the neurobiological basis of suicidal ideation. Given the previously described differences in immune-inflammatory and HPA dysregulation markers between suicide attempters and depressed and non-depressed controls, looking into associations with suicidal ideation and its severity in these three groups might help to understand the condition-specific and global differences.

Identifying biomarkers of suicidal ideation could help in the early identification and prevention of suicidal behaviours and suicide. Previous studies have identified several potential biomarkers of suicidal ideation, including cortisol, inflammatory markers, and neurotrophic factors [28,29]. However, most of these studies have focused on steroids like cortisol, immune-inflammatory proteins and blood cells [10,30], so the potential role of other biomarkers, such as peptides like ACTH, remains unclear. Examining the relationship between ACTH and other potential biomarkers of suicidal ideation may provide a more comprehensive understanding of the neurobiology of the HPA axis and its influence on suicidal ideation. Therefore, this study aimed to investigate the association between peripheral ACTH, cortisol, inflammatory cytokine and other biomarker levels and suicidal ideation in patients with major depression disorder (MDD), and patients with a recent suicide attempt, in comparison to healthy controls.

## 2 Methods

### 2.1 Study participants

The study is a case-control observational multi-centric study conducted in three major hospitals in Vilnius City, Lithuania, between June 2020 and June 2021. The study recruited a total of 156 individuals into three groups of equal size: suicide attempters, individuals with a MDD without a history of a suicide attempt, and non-psychiatric controls (volunteers currently not suffering from any mental disorder). The rationale for including people with MDD and also suicide attempters was that suicidal ideation can be conceptualized as a symptom of a major depressive episode, for which it is a diagnostic criterion, and as part of a separate clinical phenomenon of STB with its own risk factors, progression and onset, since not all individuals who die by suicide are depressed and vice versa. Inclusion criteria comprised of being an adult and having the capacity to give informed consent, while exclusion criteria included currently suffering from a drug-induced mental illness, from a severe somatic illness requiring intensive treatment, not understanding the national language, being deprived of liberty or having reduced legal capacity, and being unable to understand and sign the informed consent form.

The study was approved by the Vilnius Regional Biomedical Research Ethics Committee of Lithuania (No.2020/9-1266-747), and all procedures were in accordance with the Helsinki Declaration and its later amendments.

### 2.2 Patient assessment

Patients with MDD and healthy controls were screened for past SA, but not past SI. Suicide attempters were not screened for psychiatric disorders, as they were recruited in a General Hospital Emergency Department by its personnel, and did not undergo a throughout psychiatric evaluation. The information about antidepressant use was collected in all groups. To measure depression severity, we used the Hamilton Depression Rating Scale (HDRS-17) [31], which is a 17-item interviewer-administered measure of depressive symptom severity over the past week. The Beck Scale for Suicide Ideation/Suicide Severity Index (BSS/SSI) [32], which is a 19-item self-report instrument for detecting and measuring the intensity of attitudes, behaviours, and plans to attempt suicide over the past week, was used to measure SI severity. The study also collected information about alcohol use, smoking status, and sociodemographic variables including the length of education, income, and relationship status. Given that suicide attempters often underreport alcohol use [33], plasma phosphatidylethanol (PEth) level was used to measure alcohol exposure.

### 2.3 Biological analytes

To measure immune-inflammatory and stress response system-related markers, fasting blood samples were taken in all individuals in the morning in endotoxin-free 3.8 % sodium citrate tubes (Vacutainer®, Becton-Dickinson) within 24 h after the initial evaluation. We measured plasma levels of HPA markers ACTH, cortisol, immune-inflammatory markers IL-6, TNF-alpha, CRP, and steroid hormone DHEA, which enhances immunity and has anti-glucocorticoid effects [34].

### 2.4 Statistical analysis

The raw data was initially examined through a series of tests to confirm its normal distribution, identify any anomalies or errors, and verify its credibility. The Shapiro-Wilk and Levene's tests were used to assess the normality and homogeneity of the continuous data. Since data showed a non-Gaussian, distribution, non-parametric analysis methods were used to compare the distribution of continuous variables between the groups: Kruskal Wallis H test was used to compare the three groups and the chi-square test was used to calculate the difference between groups where variables were nominal. Kendall partial rank correlation was used for correlation analyses.

To examine the association between biomarkers and suicidal behaviour, multiple multivariate regression models were run and then adjusted for significant confounders. The following confounders were included in the analyses: age, sex, BMI, smoking status, plasma Peth concentrations, education status, antidepressant use, and plasma CRP levels. Residuals in regression models were checked and probability plots were visually examined. The Variance Inflation Factor (VIF) statistic was used to check for the presence or absence of collinearity. The significance level was set at a two-sided p-value of 0.05, obtained with 2000 sample bootstrapping. All analyses were performed using the Statistical Package for the Social Sciences (SPSS) software, version 28.

## 3 Results

The sample consisted of 156 individuals (females = 108, 69 %), among which 51 had a recent SA, 52 had a MDD without a history of SA (103 patients in total), and 53 were non-psychiatric controls.

Significant differences between the suicide attempters, patients with MDD and controls were detected in the following variables: age, education, antidepressant use, DHEA levels, income, diastolic blood pressure, heart rate, HAM-D total score, SSI total score, history of past episode of depression, SA history, family history of depression, current smoking, plasma CRP levels, plasma TNF-alpha levels, and self-reported alcohol use.

### 3.1 Biomarker concentration analysis

TNF-alpha levels were significantly lower in the suicide attempt group (4.18 ± 2.30, than in the depression group (5.33 ± 2.06) and control group 4.72 ± 2.25 (p < 0.001). They were able to predict suicidal ideation in the linear regression model, but in an inverse direction – higher TNFalpha was predictive of lower suicidal ideation.

With IL6, we have found a classical relationship, that higher levels of IL6 were observed in suicide attempt patients (6.56 ± 6.90), depression group (4.43 ± 3.39) vs 3.46 ± 1.28 in controls, however, the significance level was not achieved (p = 0.34).

CRP levels were much higher both in suicide attempters (12.48 ± 28.42) and the depression group (2.98 ± 4.03) vs. (1.41 ± 2.36) in controls (p < 0.001).

ACTH levels were significantly different between groups – 28.14 ± 15.89 in the depression group, 21.13 ± 16.85 in the suicide attempt group and 24.12 ± 14.76 in the control group (p = 0.023).

Cortisol levels were not significantly different between the three groups – 403.95 ± 111.77 in the depression group, 351.08 in the suicide attempt group, and 384.19 in the control group (p = 0.122). DHEA levels were lower in both depression (6.45 ± 3.26) and suicide attempt groups (6.60 ± 5.30) than in controls (7.82 ± 4.00), but the difference was not statistically significant (p = 0.066).

**Fig. 1:**
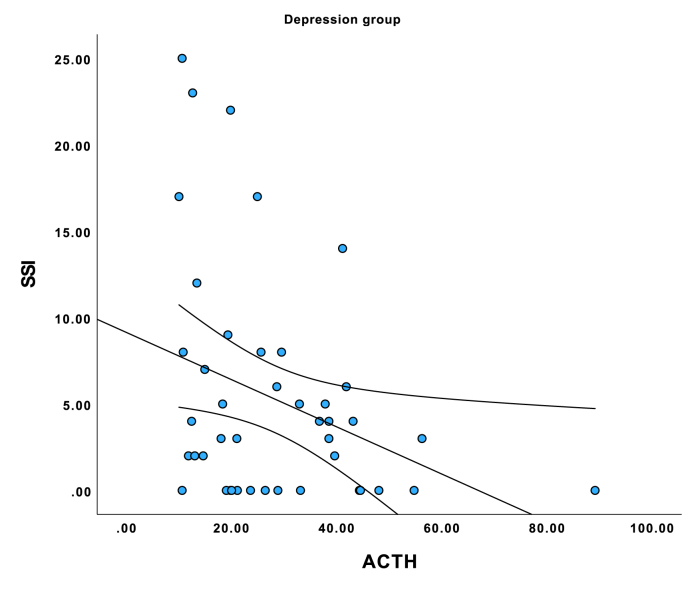
Correlation between SSI score and plasma ACTH levels in major depressive disorder group.

**Fig. 2:**
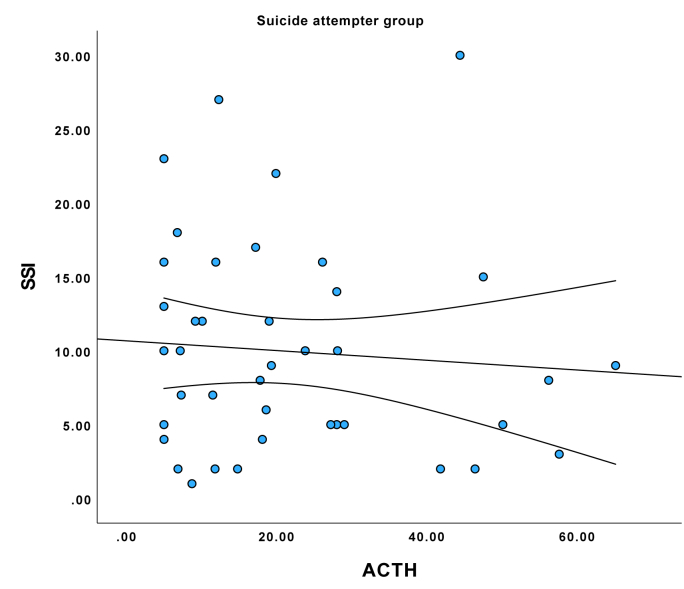
Correlation between SSI score and plasma ACTH levels in suicide attempter group.

**Fig. 3:**
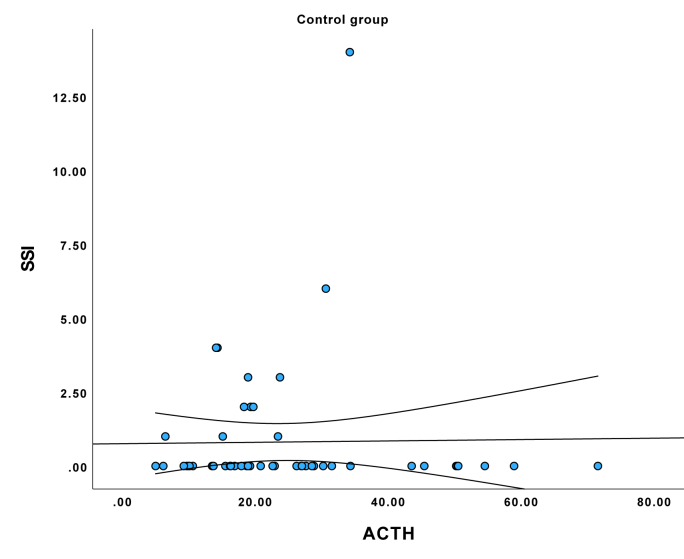
Correlation between SSI score and plasma ACTH levels in control group.

See comparisons in Table 1. (See Fig. 1, Fig. 2, Fig. 3)

**Table 1:** Sample description.

Variable	Mean or SD if % Depression Group		Mean or SD if % Suicide attempters Group		Mean or SD if % Control group		df	KWH	P (kwh/chi^2^)
***SOCIODEMOGRAPHIC DATA***									
Sex, female %	75 %		66 %		66 %		2	1.216	0.545
Age, years	39.75	13.24	41.63	19.81	33.49	12.77	2	5.773	0.056
**Education, years**	**15.1**	**3.21**	**12.60**	**2.43**	**18.80**	**2.66**	**2**	**64.13**	**<0.001**
In a relationship	36.5 %		42.9 %		53.8 %		2	3.18	0.202
**Net income, EUR per month**	**1018.18**	**757.96**	**851.72**	**723.64**	**2195.63**	**1328.37**	**2**	**39.76**	**<0.001**
**Smoking status, current smoker**	**42.3 %**	**..**	**38.8 %**	**..**	**5.8 %**	**..**	**2**	**20.34**	**<0.001**
**Self-reported alcohol use**	**60.8 %**		**52.8 %**		**79.2 %**		**2**	**7.48**	**0.024**
**Objective alcohol use (Peth)**	**58.87**	**135.61**	**233.23**	**393.60**	**25.67**	**70.43**	**2**	**12.23**	**0.002**
**Discordance between self-report and actual use**	**0 %**	**..**	**15.7 %**	**..**	**9.4 %**	**..**	**2**	**8.37**	**0.015**
BMI	26.00	6.39	24.09	4.39	23.64	5.18	2	2.82	0.244
Systolic blood pressure	122.54	14.64	127.57	15.88	121.88	14.77	2	3.95	0.140
Diastolic blood pressure	79.90	9.64	80.70	76.76	10.19		2	4.48	0.110
**Heart rate per minute**	**78.37**	**9.99**	**84.37**	**14.39**	**70.19**	**7.70**	**2**	**27.83**	**<0.001**
***CLINICAL DATA***									
Recent Stressful events	46 %	.	39 %	.	0 %	.	2	1.22	0.550
**History of depression**	**75 %**	**..**	**61 %**	**..**	**4 %**	**..**	**2**	**59.72**	**<0.001**
**History of suicide attempt**	**19 %**	**..**	**55 %**	**..**	**0 %**	**..**	**2**	**43.35**	**<0.001**
**Family history of depression**	**46 %**	**..**	**25 %**	**..**	**17 %**	**..**	**2**	**11.25**	**0.004**
**Family history of suicide attempt**	**27 %**	**..**	**8 %**	**..**	**8 %**	**..**	**2**	**10.52**	**0.005**
**Antidepressant use**	**96 %**		**59 %**	**..**	**0 %**	**..**	**2**	**98.23**	**<0.001**
**HAM-D score**	**19.72**	**5.14**	**16.25**	**5.23**	**3.81**	**4.37**	**2**	**96.49**	**<0.001**
**SSI score**	**4.81**	**6.40**	**9.92**	**6.73**	**0.81**	**2.24**	**2**	**73.53**	**<0.001**
***BIOLOGICAL MARKERS***									
ACTH	28.14	15.89	21.13	16.85	24.12	14.76	2	7.59	0.023
Cortisol	403.96	111.77	351.08	134.54	384.19	129.04	2	4.21	0.122
DHEA	6.45	3.26	6.59	5.29	7.82	4.00	2	5.45	0.066
**CRP**	**2.98**	**4.03**	**12.48**	**28.42**	**1.41**	**2.36**	**2**	**14.61**	**<0.001**
IL-6	4.43	3.39	6.56	6.90	3.45	1.28	2	2.17	0.34
**TNF-alpha**	**5.33**	**2.06**	**4.18**	**2.30**	**6.59**	**3.14**	**2**	**17.22**	**<0.001**

Abbreviations: ACTH, adrenocorticotropic hormone; BMI, body mass index; CRP, C-reactive protein; DHEA, dehydroepiandrosterone; HAM-D – Hamilton depression rating scale; IL-6 – interleukin-6; PEth – phosphatidylethanol; SA, suicide attempt; SSI, suicide severity index; TNF-alpha, tumor necrosis factor-Alpha Tests used – Chi2 and Kruskal Wallis H, p values are two sided.

Since the MDD and suicide attempters’ groups were comparable for many variables, they were combined for post-hoc analyses looking at differences between patients (attempters or MDD) versus controls (see Supplemental Table 1).

### 3.2 Correlation between peripheral blood biomarker concentrations ACTH and SSI

In the total sample, there was a positive association between ACTH and TNF-alpha (tau = 0.146, p = 0.029), cortisol (tau = 0.363, p < 0.001), and DHEA (tau = 0.138, p = 0.020). Regarding associations with SI severity, we found a negative correlation between ACTH and SSI total score (tau = −0.130, p = 0.033). SSI score was negatively correlated with TNF-alpha (tau = −0.231; p < 0.001), positively correlated with IL-6 (tau = 0.154, p = 0.015), and CRP levels (tau = 0.153, p = 0.015). In the total sample, plasma ACTH concentration did not correlate with depression severity (tau = 0.022, p = 0.711).

Correlation analysis stratified in three groups showed that ACTH was correlated negatively with SSI total score in the MDD group (tau = −0.237, p = 0.031), but not in suicide attempters (tau = −0.077, p = 0.485) or controls (tau = −0.038, p = 0.729). There were no correlations between SSI, TNF-alpha, and CRP levels in neither group in group-stratified analyses, but IL6 was positively correlated with SSI in the depression group (tau = 0.229, p = 0.039). In the patient group combining MDD and suicide attempters, ACTH concentration correlated negatively with SSI score (tau = −0.215, p = 0.005). Group-stratified analyses did not find associations between biomarker levels and depression severity in any of the groups.

In the analysis additionally adjusted for depression severity, ACTH remained associated with SI severity in the MDD group (F = 5.58, p = 0.023), and in a combined patient group (F = 5.68, p = 0.019), but not in control and SA groups.

### 3.3 Multivariate regression analyses

Lastly, multivariate linear regression models were performed. Given that ACTH emerged as the biomarker associated with SI severity, further multivariate analyses the robustness of this association after confounder adjustment. In the analysis adjusted for age, sex, BMI, education, smoking status, CRP, PEth concentration (a measure of alcohol exposure), and antidepressant use, ACTH concentration remained negatively associated with SSI score (t = −2.44, p = 0.019) in the patient group, while it was not the case in the control group (t = 0.36, p = 0.021). It also remained associated with SSI score in the MDD group (t = −2.71, p = 0.011), but not in suicide attempters (t = −0.37, p = 0.721) group.

In a subsequent regression model additionally adjusted for depression severity, plasma ACHT levels were still associated with SSI score in the combined patient group (t = −2.40, p = 0.021) and MDD group (t = −2.99, p = 0.006), but not in suicide attempters (t = −0.63, p = 0.551), or healthy controls (t = −0.99, p = 0.327). See Table 2 for further details.

**Table 2:** Association between plasma ACTH levels and suicidal ideation severity: multivariate linear regression.

Study group	Model 1			Model 2			Model 3		
	Standardized beta	T	P	Standardized beta	T	P	Standardized beta	T	P
**Total sample**	−0.69	−1.95	0.053	−0.06	−1.68	0.097	**−0.08**	**−2.36**	0.021*
**MDD group**	**−0.14**	**−2.23**	0.031*	**−0.17**	**−2.71**	0.011*	**−0.18**	**−2.99**	0.006*
**SA group**	−0.033	−0.50	0.618	−0.08	−0.37	0.721	−0.09	−0.63	0.551
**Combined patient group**	**−0.109**	**−2.40**	0.019*	**−0.16**	**−2.44**	0.019*	**−0.195**	**−2.40**	0.021*
**Control group**	0.02	0.109	0.913	0.009	0.36	0.721	−0.16	−0.99	0.327

Model 1: crude associations.

Model 2: adjusted for age, sex, education years, body mass index, smoking status, plasma CRP concentration, plasma PEth concentration (measuring chronic alcohol exposure), and antidepressant use.

Model 3: model 1 additionally adjusted for depression severity.

*p < 0.05, two-sided.

Lastly, a linear regression model was performed where all the significant variables from Table 1 were entered as covariates. ACTH remained statistically significantly associated with SSI score in the combined patient group (t = −2.41, p = 0.026), and the MDD group (t = −2.79, p = 0.016), but not suicide attempters’ and control groups.

## 4 Discussion

In this study, we studied associations of peripheral concentrations of selected stress-response and immune-inflammation system markers with suicidal ideation in recent suicide attempters, patients with MDD, and healthy controls. We report an association between plasma ACTH levels and the severity of SI in patients with MDD, but not in recent suicide attempters, which included patients irrelevant to their mental health disorders, or healthy controls. This association was robust to adjustment for possible confounders, including antidepressant use and current depression severity.

HPA axis impairment link to STB is a topic of wide attention. However, data on the ACTH is lacking, as compared to a better-researcher cortisol [35,36]. A few studies on ACTH in psychiatric populations are older than ten years, and there is a dearth of new research [37,38]. Some studies reported a connection between ACTH and SI [39,40], while others found no strong associations (B. [41,42]). Indirect evidence supporting the interest of ACTH in STB comes from the fact that lithium, the best-known agent associated with decreased risk of suicidal behaviours, increases ACTH response to dexamethasone-suppressed CRH test [43,44].

Inflammation has gained increasing traction in psychiatric research and has been proposed to be associated with STB, among other psychiatric symptoms and phenomena [45,46]. Some experts have proposed to investigate systemic anti-inflammatory therapies working on the HPA axis to treat mental health conditions [47]. Cortisol is one of the most potent anti-inflammatory agents known, and synthetic drugs mimicking cortisol are used widely to treat inflammatory conditions in other areas of medicine. However, the use of these types of medications, like prednisolone, in psychiatry is problematic due to their side effects like psychosis risk, aggravation of depression, and metabolic side effects. Cortisol itself has been associated with an increased risk of STB in numerous studies, although whether this is a direct effect or this is a marker of broad dysregulation is not clear [15,[48], [49], [50]]. Our finding about ACTH proposes an interesting alternative. Chronic stress downregulates ACTH production due to the feedback loop, and, as a consequence, people then have diminished cortisol response due to acute stressful situations.

Even less studied is the DHEA, although some studies found relationships between DHEA levels and depression [51,52]. While we found that DHEA levels were lower in psychiatric patients than controls with no differences between MDD patients and suicide attempters, it was not associated with SI severity. A recent meta-analysis found that DHEA was related to SA history [53]. For instance, a study on US veterans found a significant relationship between DHEA levels and STB [54], although the veteran population is specific due to the fact of a high prevalence of PTSD which distorts the HPA axis [55]. Another study found that DHEAS levels were higher in male, but not female, suicide attempters, compared to healthy volunteers [56]. On the other hand, a longitudinal study found no effects between androgens and suicidality [57]. These ambivalent results indicate that more research is necessary on the role of DHEA and its peripheral concentrations in STB. Our finding, that DHEA was the only steroid molecule higher in the control group than in the patient group further complicates our understanding of DHEA's role in STB.

When looking at the concentration analyses of immune-inflammatory biomarkers we found some interesting results. First, we found that CRP and IL-6 were the highest in suicide attempters, followed by MDD patients and controls, corresponding to the increasing literature suggesting increased levels of both of these molecules in people with STB [45,46,58,59]. While we did not find associations between levels of these markers with SI, the literature suggests that associations with SA seem to be more robust than with SI for these markers [6,10].

We found that TNF-alpha was negatively correlated with SI severity. Notably, TNF-alpha has both pro-inflammatory and anti-inflammatory effects [[60], [61], [62]], and its role in mental health is still somewhat controversial. Some previous studies have described a positive relationship between TNF-alpha and depression [63,64]. In addition, TNF-alpha polymorphisms have been associated with an earlier age of first SA in individuals with schizophrenia [65] and with SA in depressed patients (Y. K [74]). However, a cumulative meta-analysis that investigated cytokines in patients with MDD found a clear signal for CRP, and IL-6, but an uncertain effect of TNF-alpha due to significant heterogeneity in study-specific estimates and inconsistencies between subgroups [66]. Another study that investigated associations of three major physiological stress systems with SI found clear associations with IL-6 and CRP, but not TNF-alpha [46]. Recent studies and data syntheses found no associations between plasma concentrations of TNF-alpha and STB in people with depression [10,67].

Divergent results for TNF alpha in suicidal behaviour were reported by another major study [68]. These findings suggest that the relationship between TNF alpha and suicidality may be complex and warrant further investigation. Also, the fact that some patients were using antidepressants can confound the situation as well. Lastly, another intriguing hypothesis is that past studies found a positive relationship between TNF-alpha levels and depression, but not suicidal ideation, the same as we have found in this study with ACTH. This further solidifies the view that depression and suicidality, although related, are somewhat distinct processes molecularly.

We found a positive association between antidepressant use and SI severity. However, one should not make a false assumption that antidepressants increase it, but rather that this is confounding by indication because people with suicidal ideation are treated with antidepressants. Rigorous studies investigating antidepressant use and suicidality did not find any negative influence after adjusting for underlying disease [69,70]. Antidepressants when used long-term are useful treatments for mental health conditions like suicidal ideation [71], even though they do not work directly on the HPA axis. However, they do affect it by indirect mechanism, most likely through the monoamine system [72,[73], [74]]. For example, imipramine was shown to normalise ACTH in depressive and anxiety behaviour studies [75]. However, other studies criticise current antidepressants for not working directly on the HPA axis. A meta-analysis of 39 studies found that the more marked depressed patients' alterations in the HPA axis, the less efficacious the antidepressants [76].

The association between lower ACTH levels and SI severity in MDD (but not in recent suicide attempters) might help to elucidate the pathophysiology of SI in people suffering from SI but not attempting suicide from those who attempt suicide. Interestingly ACTH levels were higher in MDD patients compared to suicide attempters and were negatively associated with SI in MDD, suggesting that higher ACTH levels might be protective against STB at least in some patients. While further studies should aim to replicate these findings, our findings suggest that low ACTH might be implicated in STB. In the future, interventions targeting ACTH levels should be explored in people at risk of STB. On the other hand, morning plasma ACTH concentration might become a biomarker of SI severity, potentially helping to detect people in need of more intense care, as well as showing an objective marker for patients needing psychoeducation regarding the need for medical or psychological interventions.

## 5 Strengths and limitations

This study has several notable strengths and limitations. The three-group approach allowed us to disentangle associations between SI and biological markers in different patient and non-patient groups, allowing us to detect an MDD-specific association. For the study purposes, we employed consistent procedures and clinical interviews with the same methodology and rigour across all sites, ensuring data quality and consistency. However, to capture recent suicide attempters, we recruited suicide attempters in a general hospital, and study investigators were trained to administer only depression and suicide severity scales, but not to make psychiatric diagnoses. This meant that the patients did not undergo an extended psychiatric evaluation, and psychiatric comorbidities and precise diagnoses could not be assessed. The sample size was limited, which may affect the generalizability of the findings. Although we made efforts to ensure data quality and consistency, the observational nature of the study means that no causal relationships can be drawn. Furthermore, we relied on self-reported sociodemographic and clinical data, which may be subject to response bias. The lack of ethnic diversity and non-inclusion of minors in the sample is another limitation that should be noted.

## 6 Conclusions

In conclusion, higher plasma levels of ACTH were associated with lower levels of SI in patients with MDD, but not in recent suicide attempters or healthy controls. This association remained significant even when controlling for antidepressant use and depression severity. These findings further reinforce the importance of the HPA axis and, notably, ACTH on SI in patients with MDD and prompt further research of potential antidepressants that would work on the HPA axis directly. While this study provides insights into the relationship between ACTH and psychiatric psychopathology, caution should be exercised when interpreting the results due to the study's limitations, and the results need to be replicated in well-designed larger samples, ideally with a longitudinal approach.

## Statements and declarations

The authors have nothing to state or declare.

## Role of the funding source

The work was partly funded by the Faculty of Medicine, Vilnius University.

## CRediT authorship contribution statement

**Robertas Strumila:** Conceptualization, Data curation, Formal analysis, Investigation, Methodology, Project administration, Writing – original draft, Writing – review & editing. **Aiste Lengvenyte:** Conceptualization, Data curation, Project administration, Supervision, Validation, Visualization, Writing – review & editing. **Linas Zdanavicius:** Methodology. **Robertas Badaras:** Conceptualization, Data curation. **Edgaras Dlugauskas:** Conceptualization, Data curation, Methodology. **Sigita Lesinskiene:** Funding acquisition, Resources, Supervision. **Eimantas Matiekus:** Data curation, Project administration. **Martynas Marcinkevicius:** Conceptualization, Funding acquisition, Project administration, Resources. **Lina Venceviciene:** Data curation, Investigation. **Algirdas Utkus:** Conceptualization, Project administration, Resources, Supervision. **Andrius Kaminskas:** Investigation, Software. **Tomas Petrenas:** Investigation, Software. **Jurgita Songailiene:** Conceptualization, Data curation, Investigation, Supervision. **Dalius Vitkus:** Investigation, Software. **Laima Ambrozaityte:** Conceptualization, Methodology, Project administration, Supervision, Validation.

## Declaration of competing interest

The authors declare that they have no known competing financial interests or personal relationships that could have appeared to influence the work reported in this paper.
