# Supplementary material for: Higher levels of plasma Adrenocorticotropic hormone (ACTH) are associated with lower suicidal ideation in depressed patients compared to controls and suicide attempters, independently from depression severity
Source: Compr Psychoneuroendocrinol. 2024 Apr 26;19:100235. doi: 10.1016/j.cpnec.2024.100235 (PMC11087924; doi:10.1016/j.cpnec.2024.100235)
Supplement: Multimedia component 1 [file mmc1.docx]

| Variable | Mean or %  patients | SD (if mean) | Mean or %  controls | SD (if mean) | Z score | MWU | P (mwu) |
| --- | --- | --- | --- | --- | --- | --- | --- |
| **Sociodemographic data** | | | | | | | |
| Sex, female | 71% |  | 66% |  | -.618 | 2597.500 | .537 |
| **Age, years** | **40.68** | **16.76** | **33.49** | **12.77** | **-2.374** | **2095.500** | **.018** |
| **Education, years** | **13.98** | **12.77** | **18.80** | **2.66** | **-7.265** | **606.000** | **<.001** |
| In a relationship | 39% | - | 54% | - | -1.698 | 1766.000 | .089 |
| **Net income, eur per month** | **957.84** | **745.45** | **2195.63** | **1328.37** | **-6.204** | **661.500** | **<.001** |
| **Smoking status, current smoker** | **41%** | **-** | **6%** | **-** | **-4.493** | **1711.500** | **<.001** |
| **Self-reported alcohol use** | **57%** | **-** | **79%** | **-** | **-2.623** | **1803.500** | **.009** |
| BMI | 25.30 | 5.78 | 23.64 | 5.18 | -1.171 | 1838.000 | .242 |
| Systolic blood pressure | 124.79 | 15.33 | 121.88 | 14.77 | -1.464 | 1963.500 | .143 |
| **Diastolic blood pressure** | **80.20** | **9.60** | **76.76** | **10.19** | **-2.109** | **1577.000** | **.035** |
| **Heart rate per minute** | **80.56** | **12.06** | **70.19** | **7.71** | **-5.079** | **922.500** | **<.001** |
| **Clinical data** |  |  |  |  |  |  |  |
| Stressful events | 43% | - | 0% | - | -.861 | 26.500 | .389 |
| **History of depression** | **68%** | **-** | **4%** | **-** | **-7.592** | **977.500** | **<.001** |
| **History of suicide attempt** | **37%** | **-** | **0%** | **-** | **-5.068** | **1722.500** | **<.001** |
| **Family history of depression** | **36%** | **-** | **17%** | **-** | **-2.449** | **2212.500** | **.014** |
| Family history of suicide attempt | **17%** | - | **8%** | - | -1.682 | 2458.500 | .093 |
| **Antidepressant use** | **78%** | **-** | **0%** | **-** | **-9.163** | **609.500** | **<.001** |
| **HAM-D score** | **18.00** | **5.44** | **3.81** | **4.37** | **-9.588** | **170.500** | **<.001** |
| **SSI score** | **7.34** | **7.02** | **0.81** | **2.25** | **-7.279** | **844.500** | **<.001** |
| **Biological markers** |  |  |  |  |  |  |  |
| ACTH | 24.72 | 16.64 | 24.11 | 14.76 | -.059 | 2265.500 | .953 |
| Cortisol | 380.10 | 124.63 | 384.19 | 129.04 | -.536 | 2054.000 | .592 |
| **DHEA** | **6.52** | **4.29** | **7.82** | **4.00** | **-2.252** | **1630.000** | **.024** |
| **CRP** | 7.37 | **19.99** | 1.41 | **2.36** | **-3.633** | **1329.500** | **<.001** |
| IL-6 | 5.37 | 5.31 | 3.47 | 1.28 | -1.154 | 1845.000 | .248 |
| **TNF-alpha** | **6.59** | **3.14** | **4.72** | **2.25** | **-3.684** | **944.500** | **<.001** |
| PEth | 145.2 | 304.65 | 25.68 | 70.43 | -1.205 | 2420.500 | .228 |

Abbreviations: ACTH, adrenocorticotropic hormone; BMI, body mass index; CRP, C-reactive protein; DHEA, dehydroepiandrosterone; HAM-D – Hamilton depression rating scale; IL-6 – interleukin-6; PEth – phosphatidylethanol; SA, suicide attempt; SSI, suicide severity index; TNF-alpha, tumor necrosis factor-Alpha

Chi^2^ and Mann Whitney U tests were used, p-values are two-sided.
